# Single- and multiple-dose pharmacokinetics and safety of the SARS-CoV-2 3CL protease inhibitor RAY1216: a phase 1 study in healthy participants

**DOI:** 10.1128/aac.01450-24

**Published:** 2025-01-31

**Authors:** Yue Hu, Haijun Li, Kun Wang, Dandan Wu, Hong Zhang, Yanhua Ding, Junyan Wu, Suiwen Ye, Yun Peng, Li Liu

**Affiliations:** 1 Phase I Clinical Research Center, First Hospital of Jilin University 117971 , Changchun, China; 2 Department of Anatomy and Neurobiology, School of Basic Medical Science, Central South University 618101 , Changsha, China; 3 Guangdong Raynovent Biotech Co., Ltd., Guangzhou, China; 4 Shanghai Qiangshi Information Technology Co., Ltd., Shanghai, China; 5 Phase I Clinical Trial Centre, Sun Yat-sen Memorial Hospital, Sun Yat-sen University 26469 , Guangzhou, China; 6 Department of Pediatrics, First Hospital of Jilin University 117971 , Changchun, China; IrsiCaixa Institut de Recerca de la Sida, Barcelona, Spain

**Keywords:** RAY1216, pharmacokinetics, safety, 3CL protease inhibitor, COVID-19

## Abstract

Coronavirus disease 2019, which leads to pneumonia, is caused by severe acute respiratory syndrome coronavirus 2 (SARS-CoV-2). RAY1216 is a 3C-like protease inhibitor that targets SARS-CoV-2. The aim of our study was to assess the pharmacokinetics (PK) and safety of RAY1216 in healthy volunteers. This was a randomized, placebo-controlled, double-blind study consisting of four components: a single ascending dose study, a drug-drug interaction study, a multiple ascending dose study, and a food-effect study. All participants were randomly assigned to receive either a single dose or multiple doses of RAY1216 or placebo. A total of 88 healthy adult participants (male-to-female ratio of 1:1) aged 18–50 years were enrolled. A total of 37 participants (42%) experienced at least one adverse event (AE). All AEs were mild or moderate and were resolved without additional treatment. The most commonly reported adverse drug reactions were hypertriglyceridemia, hyperuricemia, and elevated serum creatinine levels. RAY1216 was well-absorbed after administration with exposure increasing in a dose-dependent manner. Food appeared to increase exposure and delay the absorption of RAY1216. Ritonavir significantly inhibited drug metabolism, and increased drug exposure increased the associated safety risks. RAY1216 was found to be well tolerated and safe in healthy participants. On the basis of preclinical results, PK characteristics, and the safety profile of RAY1216, a dosage of 400 mg three times daily was selected, thereby establishing a foundation for future research and for the clinical application of RAY1216.

**CLINICAL TRIALS:**

This study is registered with ClinicalTrials.gov as NCT05829551.

## INTRODUCTION

Coronavirus disease 2019 (COVID-19), which is caused by severe acute respiratory syndrome coronavirus 2 (SARS-CoV-2), frequently results in pneumonia (1). As of 21 March 2023, over 700 million cases of COVID-19 have been reported across 200 countries, with over 6 million deaths reported globally. Since its emergence in 2019, COVID-19 has become one of the greatest threats to human health in the 21st century (2, 3). Therefore, there is a need to develop effective anti-COVID-19 agents. With an enhanced understanding of the mechanisms underlying viral infection, numerous anti-COVID-19 drugs have been developed; however, the efficacy of these drugs remains unsatisfactory (4–7). Currently, Pfizer’s new drug, PAXLOVID (nirmatrelvir plus ritonavir [RTV]), has received approval for the treatment of COVID-19 in the United Kingdom, Australia, the EU, the USA, and Canada. Nirmatrelvir acts as an inhibitor of the 3C-like (3CL) protease, a crucial enzyme in coronaviruses that plays a vital role in mediating viral replication and transcription (8–12). However, due to its pharmacokinetic (PK) characteristics—i.e., it is metabolized by cytochrome P450 (CYP) 3A4 enzymes—a PK enhancer is necessary. RTV is known to be the most effective cytochrome CYP3A4 inhibitor in clinical application and has been used as a boosting agent (13–17).

RAY1216 is a SARS-CoV-2 3CL protease inhibitor that was independently investigated by Guangdong Raynovent Biotech Co., Ltd. (Guangdong, China). In preclinical experiments, RAY1216 clearly inhibited various SARS-CoV-2 variants, including the wild-type, Alpha, Beta, Delta, and Omicron strains, *in vitro*. It has been shown to exhibit antiviral activities against SARS-CoV-2 variants comparable to those of PF-07321332 (nirmatrelvir). PK data from animals have indicated that RAY1216 may have a promising human PK profile without ritonavir (18–21). RAY1216 has a wide safety range based on toxicological data from rat and dog studies. In summary, preclinical studies have indicated that RAY1216 is a potential drug candidate that should be further examined in human studies.

As a crucial component of new drug development, first-in-human trials play a pivotal role. Therefore, the primary objective of this study was to obtain PK data and safety information on RAY1216 through clinical trials with healthy participants and assessments of the PK characteristics of this drug under single- and multiple-dose escalation conditions, evaluation of the impact of food and coadministration of ritonavir on PK, and determination of the drug safety profiles. The study was designed to achieve these objectives via four components: the single ascending dose (SAD) study, the drug-drug interaction (DDI) study, the multiple ascending dose (MAD) study, and the food-effect study. In the food-effect study, a comprehensive investigation of RAY1216 excretion and metabolism was conducted. In addition, drug concentrations in the blood, urine, and fecal samples were analyzed, and five metabolites were measured.

## MATERIALS AND METHODS

### Drugs and participants

RAY1216 (100 mg and 200 mg) and ritonavir (100 mg) were supplied by Guangdong Raynovent Biotech Co., Ltd. (Guangdong, China). RAY1216 received approval for clinical trials from the National Medical Products Administration in May 2022. Healthy Chinese adults (males and females) aged 18–50 years, with a body mass index (BMI) of 18 kg/m^2^–28 kg/m^2^, were recruited for this study. Participants were assessed for eligibility on the basis of medical history, vital signs, physical and laboratory examination findings, electrocardiogram (ECG) findings, chest X-ray findings, and abdominal ultrasound findings. Participants were excluded if they had a viral infection (e.g., hepatitis B/C, human immunodeficiency virus, or *Treponema pallidum* infection), a marked history of clinical illness (e.g., tumors or gastrointestinal, kidney, liver, neurological, blood, endocrine, lung, immune, psychiatric, cardiovascular, or cerebrovascular diseases), positive results from alcohol or urine drug tests, or any pathological findings during the screening.

### Study design

The present study was a multicohort, double-blind, randomized, placebo-controlled phase 1 trial designed to evaluate the tolerability, PK, and safety profile of RAY1216. It comprised four parts: a SAD study, a DDI study, a MAD study, and a food-effect study.

The preclinical animal studies revealed that the no-observed-adverse-effect level (NOAEL) in rats and dogs was 900 mg/kg/day and 600 mg/kg/day, respectively. Thus, the human equivalent dose was approximately 10,800 mg/day and 21,000 mg/day at 60 kg body weight, respectively. At a safety factor of 10, a maximum recommended starting dose of 1,000 mg was determined based on the NOAEL in rats. In a K18-hACE2 mouse model, mice were infected with the Delta variant of the novel coronavirus, and RAY1216 administered at 300  mg/kg/day significantly decreased lung viral titers and reduced virus-induced pathology (18). On the basis of preclinical findings from a previous phase 1 study of a similar target drug (i.e., nirmatrelvir), we selected 400 mg as the starting dose of RAY1216 for the SAD study. However, the PK characteristics of RAY1216, when it is administered alone in humans, remain unclear. *In vitro* assays of cytochrome P450 enzyme metabolic reaction phenotypes indicated that CYP3A is the primary metabolizing enzyme for RAY1216, and the combination of RAY1216 with ritonavir resulted in increased drug exposure. In addition, clinical studies involving commercially available drugs, such as lopinavir, nirmatrelvir, and indinavir, have demonstrated that a 100 mg dose of ritonavir can effectively increase drug exposure (13–16). Consequently, we selected a fixed dose of 100 mg of ritonavir for the present study to inhibit the metabolism of RAY1216 and increase its exposure. However, if the PK data for RAY1216 administered alone suggest that it achieves sufficient therapeutic coverage in humans, the addition of ritonavir may not be necessary. Thus, we selected 400 mg of RAY1216 as the starting dose for the SAD study when it was administered as a single agent and 200 mg of RAY1216 in combination with ritonavir. Furthermore, the maximum dose was selected to not exceed the NOAEL exposures in rats. The sample sizes for each dose group were determined based on the principle of exposing as few participants as possible to RAY1216 in order to obtain sufficient tolerability, safety, and pharmacokinetic properties. For the sake of safety, there were six participants in each group, and the probability of observing at least one such serious adverse event (SAE) is greater than 88% when the potential incidence of SAEs related to the drug is ≥30%. However, if none of the six participants reported any SAEs related to the drug after dosing, the likelihood of a potential incidence of such events exceeding 46% is less than 2.5%. The food-effect study involved patients being randomly allocated to one of two sequences (fasting and feeding) at a 1:1 ratio. The PK parameters were determined after logarithmic transformation. When the *in vivo* coefficient of variation was less than 30%, the standard deviation of the difference between fasting and feeding was less than 0.42. Therefore, 12 participants were sufficient for enrollment.

**Fig 1:**
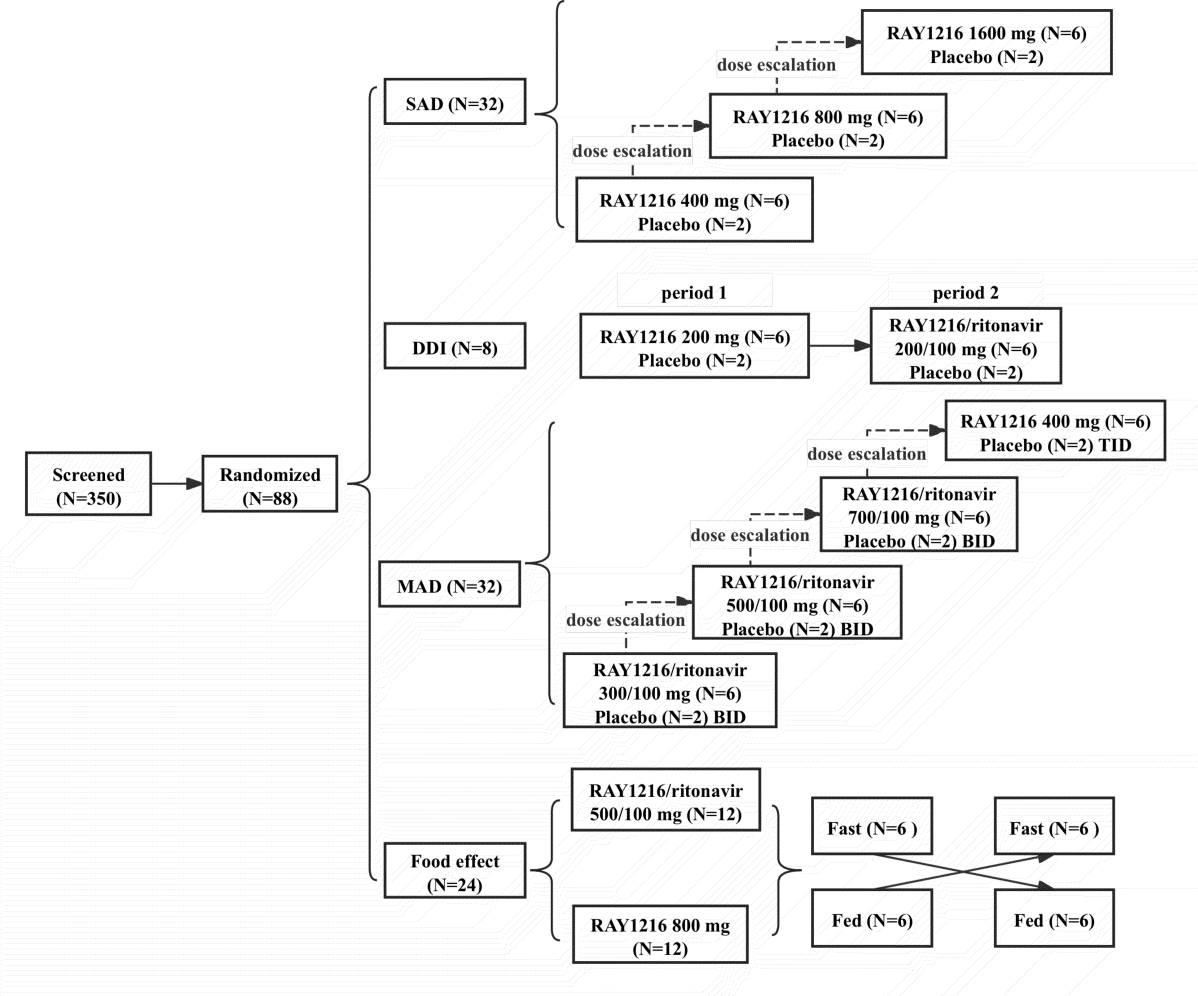
Flow diagram of different groups. A total of 350 healthy Chinese adults were screened, of which 88 healthy participants were randomized and enrolled. No participants withdrew. The SAD and MAD studies initiated from the lowest dosage, and the next highest dose level was initiated based on PK characteristics and a 5-day safety observation of the previous dose by the investigators and sponsors. For the SAD study, 400 mg of RAY1216 was the starting dose for the SAD study when it was administered as a single agent and 200 mg of RAY1216 in combination with ritonavir.

In the SAD study, three cohorts received single-dose RAY1216 at 400 mg, 800 mg, or 1,600 mg. The DDI study consisted of two periods: period 1 involved a single dose of RAY1216 at 200 mg, while period 2 involved administration in combination with ritonavir. For the MAD and food-effect studies, the doses were chosen according to the PK and safety results of the SAD study. The MAD study included four cohorts receiving RAY1216 at 300 mg twice daily (BID), 500 mg BID, 700 mg BID in combination with 100 mg of ritonavir, and 400 mg three times daily (TID) over days 1–5, with a single dose given on the morning of day 5. For the SAD and MAD studies, each cohort included eight participants (with a male-to-female ratio of 1:1), of whom six received RAY1216 and two were given the matched placebo. The tablets were administered from the lowest dosage. The next highest dose level was initiated based on PK characteristics and a 5-day safety observation of the previous dose by the investigators and sponsors. The food-effect study was a randomized, two-sequence, two-period crossover study that aimed to assess the effect of food on the PK of RAY1216 and speculation on the possible metabolic pathways of RAY1216 in the human body. This study included two groups: one group under feeding conditions (a high-fat diet comprising 110 g of boiled eggs, 240 mL of milk, 20 g of bacon, 20 g of butter, 50 g of toast, 10 g of salad oil, and 115 g of chips consumed within 30 min before dosing) and one group under fasting conditions. There were two cohorts (800 mg of RAY1216, 500 mg of RAY1216 plus 100 mg of ritonavir), and each cohort comprised 12 participants (see Fig. 1). Interventions for all cohorts, except the 200 mg cohort, were conducted at the First Hospital of Jilin University (Changchun, China). The randomization lists were created using the PLAN statistical procedure in SAS 9.4 software (SAS Institute, Cary, NC, USA). Throughout the study, unblinding was only used in the event of SAEs or other emergency situations; otherwise, blinding was maintained for investigators, participants, and sponsors until the study’s completion.

All participants were hospitalized the day prior to medication administration, and fasted for 12 h before dosing. On the day of administration, two researchers were responsible for administering the study medication at a fixed time, accompanied by 240 mL of water. Throughout the study, participants received standardized meals and abstained from smoking, alcohol, and caffeine. They voluntarily adopted effective contraceptive measures, excluding hormonal contraceptives, during the trial period. The human chorionic gonadotropin test was performed before dosing and at the final visit to assess pregnancy status.

### PK sample collection and analyses

For the SAD, food-effect, and DDI studies, blood samples were collected before dose administration and at 15 min, 30 min, and 1, 1.5, 2, 3, 4, 6, 8, 12, 24, 36, 48, 72, and 96 h after dose administration. We used collection tubes containing the anticoagulant EDTA for PK assessments. In the MAD study, blood samples were obtained on day 1 at 0 h, 15 min, 30 min, and 1, 1.5, 2, 3, 4, 6, 8, and 12 h after dosing; on days 2–4; and on day 5 at 0 h, 15 min, 30 min, and 1, 1.5, 2, 3, 4, 6, 8, 12, 24, 36, 48, 72, and 96 h after dosing. The plasma samples were subsequently centrifuged at 1,300 × *g* for 10 min at 2–8°C. For the food-effect component, urine samples were collected in tubes containing 0.01% Triton X-100 at the following intervals: 0, 0–6, 6–12, 12–24, 24–48, 48–72, 72–96, 96–120, 120–144, 144–168, and 168–192 h after administration. Fecal samples were collected from 0 h to 192 h after the first dose. All the samples were stored at −80°C.

The concentrations of RAY1216 in fecal, urine, and plasma samples were subsequently analyzed using a validated liquid chromatography‒tandem mass spectrometry (LC‒MS/MS) method. The standard curve concentrations ranged from 5 to 5,000 ng/mL, 10 to 10,000 ng/mL, and 5 to 5,000 µg/g in the plasma, urine, and fecal samples, respectively. The lower limits of quantification were 5.00 ng/mL, 10.00 ng/mL, and 5.00 µg/g for the plasma, urine, and fecal samples, respectively.

The metabolites of RAY1216 that were present in the plasma, fecal, and urine samples of healthy participants were analyzed using LC-UV-HRMS following dosing. The percentages of the parent drug and its metabolites in these samples were calculated via LC‒MS to infer the potential metabolic pathway of RAY1216 in humans.

The noncompartmental model (Phoenix WinNonlin ver. 8.2.2.227, Certara, Princeton, USA) was used to calculate the PK parameters: time to maximum observed plasma concentration (*T*_max_); maximum observed plasma concentration (*C*_max_); area under the concentration-time curve from the time of dosing to the last time point with measurable plasma concentrations of drug (AUC_0-t_); AUC from the time of dosing extrapolated to infinity (AUC_0-∞_); terminal elimination half-life of the drug in plasma (*t*_1/2_); clearance (CL/F); volume (Vz/F); terminal phase elimination rate constant (λ_z_); and mean retention time (MRT). The power model was used to evaluate the relationship between PK parameters and the dose level, expressed as ln (PK parameter) = ln (α) + β * ln (dose), where ln (α) is the intercept of the transformed linear regression equation and β is the slope. Upon calculating the 90% CI of β, if the entire 90% CI of β falls within the judgment interval, the PK parameters can be deemed linearly related to the dosage.

For the food-effect and DDI studies, a mixed-effect analysis of variance was used to compare *C*_max_ and AUC by using sequence, treatment, and period or treatment as fixed effects. The comparisons are presented in terms of geometric mean ratio and the 90% CI.

### Tolerability and safety assessment

Safety and tolerability assessments were conducted for all participants who received at least one dose of RAY1216, either alone or in combination with ritonavir, throughout the study. The safety of all participants was evaluated on the basis of clinical symptoms, vital signs, 12-lead ECG, laboratory tests, physical examinations, and the evaluation of adverse events (AEs). All AEs were documented and graded according to the Common Terminology Criteria for Adverse Events (CTCAE, ver. 5.0). The grading scale was as follows: grade 1 (mild), grade 2 (moderate), grade 3 (severe), grade 4 (life threatening), and grade 5 (death).

To evaluate the cardiotoxicity of the drug, 12-lead ECG was conducted at several time points: 0 h, 1 h, 2 h, 4 h, 24 h, 48 h, 72 h, and 96 h after dosing in the SAD, food-effect, and DDI studies. In the MAD study, 12-lead ECG data were collected on day 1 (0 h, 1 h, 2 h, 4 h, and 8 h after dosing) and on day 5 (0 h, 1 h, 2 h, 4 h, 24 h, 48 h, 72 h, and 96 h after dosing), as well as before dosing and 2 h after dosing on days 2–4. Three consecutive ECGs were performed at each of these time points. The QT interval was corrected using the Fridericia formula [QTcF = QT / (RR^0.33^), where RR = 60/heart rate]. Descriptive statistics were applied to the number of participants with abnormal changes in QTcF after dosing compared to baseline, including: (i) absolute value of QTcF >450 ms; (ii) absolute value of QTcF >500 ms; (iii) 30 ms < ΔQTcF ≤ 60 ms; (iv) ΔQTcF >60 ms (ΔQTcF = QTcF after dosing − baseline). The relationship between ΔQTcF and the concentrations of RAY1216 was explored using a linear mixed-effects model (C-QT model) (22). The C-QT model was used to predict the difference in ΔQTcF between RAY1216 and placebo (ΔΔQTcF) and to calculate the mean ΔΔQTcF and the 90% CI.

For safety analysis, we also employed an exposure-response analysis method that included four binary outcomes: any adverse drug reaction (ADR), hypertriglyceridemia, hyperuricemia, and elevated creatinine levels. The exposure levels were derived by simulating individual parameter estimates from a population PK model (23), which generated dense sampling drug concentration-time curves. The noncompartmental analysis was subsequently performed using the PKNCA package in R software ver. 4.3 to calculate the area under the concentration-time curve over 24 h (AUC_0-24_) using the trapezoidal method and the maximum blood drug concentration within 24 h (*C*_max_). Logistic regression models were used to analyze these AEs, with a significance threshold set at α = 0.05 to evaluate whether the probability of AE occurrence exhibited a significant dose-response relationship with exposure.


$$logit(P(Y_{i}=1))=\beta_{0}+\beta_{1}\cdot log(X_{i})$$

In this context, Xi represents the exposure (AUC_0-24_ and *C*_max_) of the ith subject, β_0_ denotes the intercept, and β_1_ indicates the coefficient for exposure. *P*(Yi = 1) represents the probability of AE occurrence, with logit referring to the logistic transformation.

## RESULTS

### Study participants and baseline demographics

All participants completed the study, maintaining their participation until its conclusion. The male-to-female ratio was 1:1. The demographic baseline values, including age, weight, height, and BMI, were comparable across our study groups (Table 1).

**TABLE 1:** Baseline demographic characteristics^*a*^

Cohort		Age (years)	Sex, *n* (%)		Weight (kg)	Height (cm)	Race, *n* (%) Asian
			Male	Female			
Single ascending dose							
Placebo (*n* = 8)		37.3 (9.50)	4 (50.0%)	4 (50.0%)	62.63 (10.572)	160.91 (10.004)	8 (100)
RAY1216 200 mg (*n* = 6)		28.7 (8.98)	3 (50.0%)	3 (50.0%)	61.63 (10.489)	163.42 (5.580)	6 (100)
RAY1216 400 mg (*n* = 6)		41.5 (6.35)	3 (50.0%)	3 (50.0%)	65.00 (11.552)	163.70 (14.568)	6 (100)
RAY1216 800 mg (*n* = 6)		29.7 (7.97)	3 (50.0%)	3 (50.0%)	62.02 (7.840)	166.77 (12.213)	6 (100)
RAY1216 1,600 mg (*n* = 6)		34.7 (8.41)	3 (50.0%)	3 (50.0%)	66.18 (7.962)	169.18 (14.156)	6 (100)
Food effect							
RAY1216 800 mg	Fast (*n* = 6)	32.2 (8.08)	3 (50.0%)	3 (50.0%)	61.05 (6.849)	163.75 (7.643)	6 (100)
	Fed (*n* = 6)	30.8 (7.96)	3 (50.0%)	3 (50.0%)	57.47 (5.274)	161.80 (6.534)	6 (100)
RAY1216/RTV 500 mg/100 mg	Fast (*n* = 6)	31.2 (4.07)	3 (50.0%)	3 (50.0%)	62.10 (9.998)	164.18 (9.395)	6 (100)
	Fed (*n* = 6)	34.5 (9.40)	3 (50.0%)	3 (50.0%)	57.68 (8.605)	159.87 (8.329)	6 (100)
Multiple ascending dose							
Placebo (*n* = 8)		36.8 (9.33)	3 (50.0%)	3 (50.0%)	61.73 (7.585)	165.73 (9.668)	8 (100)
RAY1216/RTV 300 mg/100 mg BID (*n* = 6)		39.2 (7.83)	3 (50.0%)	3 (50.0%)	66.15 (12.367)	164.83 (10.439)	6 (100)
RAY1216/RTV 500 mg/100 mg BID (*n* = 6)		34.8 (9.30)	3 (50.0%)	3 (50.0%)	57.23 (6.167)	163.37 (9.517)	6 (100)
RAY1216/RTV 700 mg/100 mg BID (*n* = 6)		38.0 (4.65)	3 (50.0%)	3 (50.0%)	66.73 (12.232)	167.48 (11.242)	6 (100)
RAY1216 400 mg TID (*n* = 6)		37.0 (9.65)	3 (50.0%)	3 (50.0%)	64.30 (7.316)	162.92 (10.996)	6 (100)

^*a*^ All the data are expressed as the means ± SDs, but sex and race are expressed as *n* (%). RTV, ritonavir; BID, twice daily; TID, three times daily.

### PK analysis of the SAD study

The absorption of RAY1216 was relatively rapid following a single fasting dose ranging from 200 to 1,600 mg, with a median *T*_max_ of 1.3 h–1.5 h. The average *t*_1/2_ varied between 3.25 and 10.3 h across the different cohorts. The mean values for *C*_max_, AUC_0-t_, and AUC_0-∞_ of RAY1216 were found to be 1,270 ng/mL–6,180 ng/mL, 5,510 h·ng/mL–29,100 h·ng/mL, and 5,550 h·ng/mL–29,100 h·ng/mL, respectively. The slopes of the exponential β in the linear equations for *C*_max_, AUC_0-t_, and AUC_0-∞_ were 0.7940 (90% CI: 0.6270–0.9672), 0.7817 (90% CI: 0.6330–0.9305), and 0.7797 (90% CI: 0.6312–0.9283), respectively. The judgment intervals for *C*_max_, AUC_0-t_, and AUC_0-∞_ were 0.9508–1.0494, 0.9692–1.0308, and 0.9692–1.0308, respectively. Therefore, while the *C*_max_, AUC_0-t_, and AUC_0-∞_ of RAY1216 increased in a dose-dependent manner, the linear dynamic properties of these PK parameters remain undetermined within the dosage range of 200 mg–1,600 mg (Table 2).

**TABLE 2:** PK parameters of the RAY1216 in the SAD and DDI studies^*a*^

Parameter	RAY1216 200 mg (*N* = 6)	RAY1216/RTV 200 mg/100 mg (*N* = 6)	RAY1216 400 mg (*N* = 6)	RAY1216 800 mg (*N* = 6)	RAY1216 1,600 mg (*N* = 6)
*t*_1/2_ (h)	3.25 ± 0.202	4.48 ± 0.298	4.83 ± 2.86	10.3 ± 9.72	5.22 ± 1.16
*T*_max_ (h)	1.5 (0.5, 2.0)	2.0 (1.5, 3.0)	1.3 (1.0, 4.0)	1.5 (1.0, 3.0)	1.5 (1.0, 4.0)
*C*_max_ (ng/mL)	1,270 (42)	3,270 (22.2)	2,250 (53.8)	3,590 (20.2)	6,180 (20.1)
AUC_0-t_ (ng·h/mL)	5,510 (22.7)	24,600 (18.7)	11,700 (45.8)	16,200 (18.7)	29,100 (24.1)
AUC_0-∞_ (ng·h/mL)	5,550 (22.7)	24,700 (18.6)	11,700 (45.6)	16,300 (18.8)	29,100 (24.1)
CL/F (L/h)	37.7 (24.3)	8.35 (18.8)	44.3 (62.1)	50.5 (16.9)	57.4 (22.3)
λ_z_ (h^−1^)	0.214 (6.2)	0.155 (6.6)	0.172 (36)	0.128 (73.6)	0.139 (22.4)
V_z_/F (L)	177 (26.1)	54.4 (23.6)	287 (61.3)	709 (95.9)	438 (33.7)
MRT_0-∞_ (h)	5.01 (13.5)	6.60 (10.4)	6.05 (9.9)	6.75 (38.2)	5.94 (13.4)

^*a*^ The PK parameter means (%CV) are presented except for *t*_1/2_ (mean and SD) and *T*_max_ (median and range). RTV, ritonavir.

Compared with those of 200 mg RAY1216 alone, the mean *C*_max_, AUC_0-t_, and AUC_0-∞_ increased to 348.03%, 350.07%, and 171.00%, respectively, in combination with ritonavir. Additionally, the mean *t*_1/2_ was prolonged by 1.23 h, whereas the mean CL/F was reduced by 29.35 L/h. Furthermore, relative to 200 mg RAY1216 alone, the mean *C*_12h_ concentration increased by 417.3 ng/mL. The geometric average ratios of *C*_max_, AUC_0-t_, and AUC_0-∞_ in combination with ritonavir were elevated to 271.00% (90% CI: 191.12%–384.25%), 450.07% (90% CI: 359.47%–563.51%), and 448.03% (90% CI: 358.07%–560.58%), respectively. These findings suggest that ritonavir significantly inhibits drug metabolism and significantly enhances exposure to RAY1216 as a PK enhancer (see Table 2).

### PK evaluation of the food-effect study

In the cohort administered 800 mg of RAY1216, the *T*_max_ was delayed by 1.5 h in the feeding condition compared with the fasting condition. In addition, the geometric mean ratios of *C*_max_, AUC_0-t_, and AUC_0-∞_ increased to 182.88% (90% CI: 145.25%–230.25%), 156.02% (90% CI: 133.44%–182.42%), and 154.62% (90% CI: 132.56%–180.36%), respectively.

**Fig 2:**
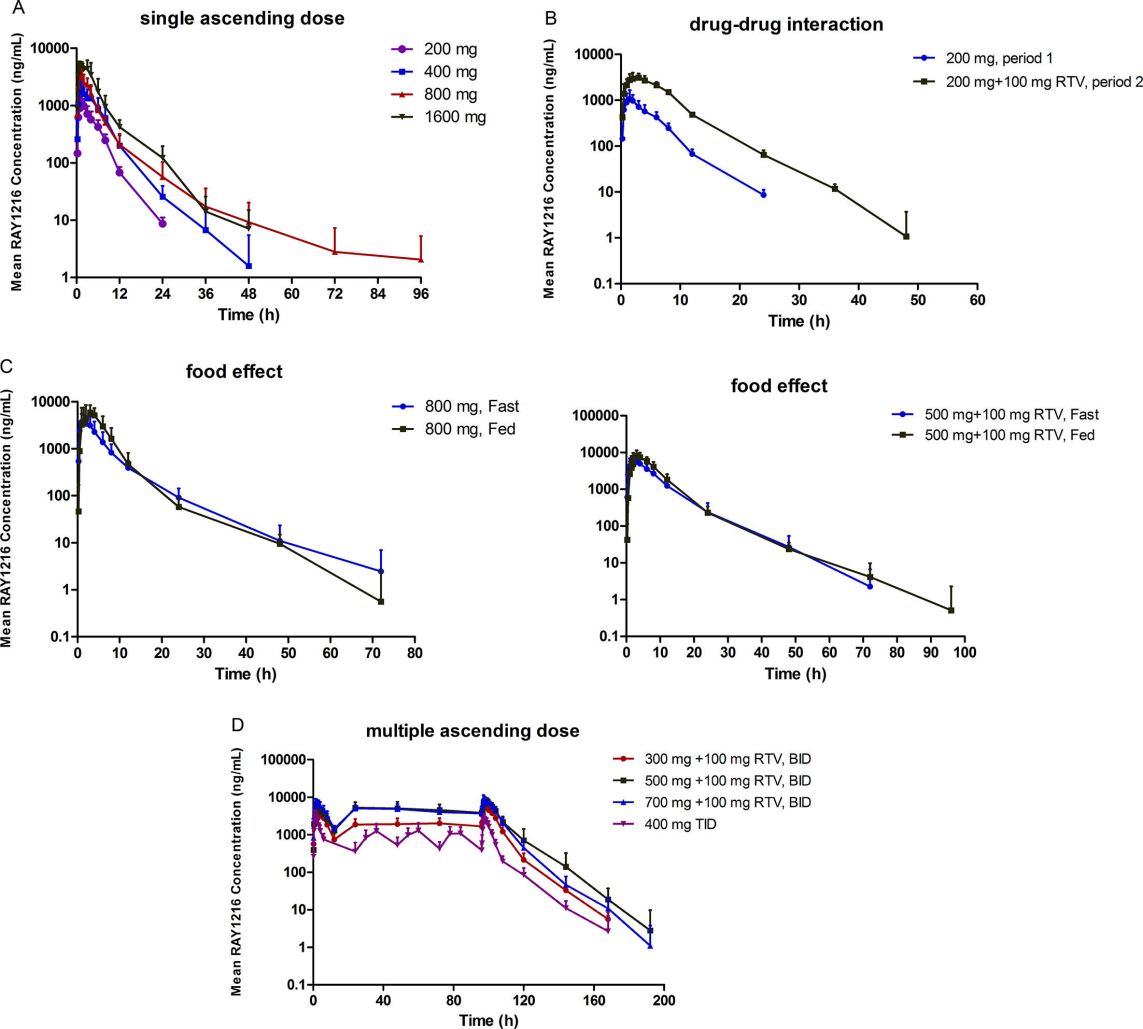
PK characteristics of RAY1216 in healthy participants, as shown in the (**A**) SAD study (RAY1216), (**B**) single-dose and DDI study (RAY1216 200 mg on day 1, RAY1216/RTV 200 mg/100 mg on day 9), (**C**) food-effect study (left represents RAY1216 800 mg alone; right shows RAY1216/RTV 500 mg/100 mg), and (**D**) MAD study (RAY1216 from day 1 to day 5).

In the cohort administered 500 mg of RAY1216 alongside 100 mg of ritonavir, the intake of high-fat food delayed the *T*_max_ of RAY1216 from 2.5 h to 3.0 h. Meanwhile, the geometric mean ratios for *C*_max_, AUC_0-t_, and AUC_0-∞_ in this cohort increased to 149.16% (90% CI: 114.81%–193.81%), 137.52% (90% CI: 107.95%–175.19%), and 136.90% (90% CI: 107.63%–174.13%), respectively, compared with the fasting condition. These findings suggest that a high-fat diet may increase plasma exposure and delay the absorption of RAY1216 (see Table 3; Fig. 2C).

**TABLE 3:** PK parameters of the RAY1216 in the food effect study^*a*^

Parameter	RAY1216 800 mg		RAY1216/RTV 200 mg/100 mg	
	Fast (*N* = 12)	Fed (*N* = 12)	Fast (*N* = 12)	Fed (*N* = 12)
*t*_1/2_ (h)	7.72 ± 4.46	7.08 ± 3.61	6.03 ± 2.42	7.83 ± 4.13
*T*_max_ (h)	1.5 (0.5, 3.0)	3.0 (1.0, 6.0)	2.5 (1.0, 4.0)	3.0 (1.5, 6.0)
*C*_max_ (ng/mL)	4,760 (31.6)	8,510 (27.6)	6,070 (35.4)	9,010 (35.1)
AUC_0-t_ (ng·h/mL)	22,700 (38.9)	34,100 (28)	49,300 (39.3)	64,900 (30.1)
AUC_0-∞_ (ng·h/mL)	23,000 (38.9)	34,200 (27.9)	49,600 (39)	65,000 (30)
V_z_/F (L)	412 (47.5)	246 (51.4)	101 (53.8)	88.6 (41)
CL/F (L/h)	40.7 (46.4)	25.1 (28.4)	12.3 (57.8)	8.41 (32)
MRT_0-∞_ (h)	7.01 (26.4)	5.96 (22.2)	8.06 (13.3)	8.21 (13.6)

^*a*^ The PK parameter means (%CV) are presented except for *t*_1/2_ (mean and SD) and *T*_max_ (median and range). RTV, ritonavir.

### PK analysis of the MAD study

Following multiple administrations of RAY1216 at doses of 300 mg, 500 mg, or 700 mg in combination with 100 mg of ritonavir BID, the plasma concentrations reached a steady state by day 2, with a *T*_max_ of 1 h–2.3 h. Drug exposure tended to increase from 300 mg to 700 mg of RAY1216. However, the rate of increase in exposure gradually slowed, as evidenced by slight decreases in AUC_0-∞,ss_ and *C*_min,ss_ in the cohort receiving RAY1216 at 700 mg in combination with ritonavir 100 mg BID. The slopes of the linear equation exponential β for *C*_max_, AUC_0-tau,ss_, AUC_0-t,ss_, and AUC_0-∞,ss_ were 0.5523 (90% CI: 0.2475–0.8570), 0.5659 (90% CI: 0.2185–0.9134), 0.6175 (90% CI: 0.2058–1.0292), and 0.6160 (90% CI: 0.2046–1.0275), respectively, with values that only partially fell within the discriminant interval. The mean *C*_min,ss_ was 1,180 ng/mL–2,520 ng/mL. The cumulative ratios for drug plasma exposure, *R*_ac_AUC_ and *R*_ac_Cmax,_ were 1.35–1.80 and 1.29–1.91, respectively. These findings indicate relatively lower drug accumulation.

Following continuous administration of 400 mg of RAY1216 TID, the plasma concentration reached a steady state by day 2, with a *T*_max_ of 1.5 h. The mean *C*_min,ss_ was 382 ng/mL. The average drug accumulation ratios for *R*_ac_AUC_ and *R*_ac_Cmax_ were 1.04 and 0.812, respectively (see Table 4; Fig. 2D). These findings indicate that there was no accumulation of RAY1216 following continuous administration.

**TABLE 4:** PK parameters of the RAY1216 in the MAD study^*a*^

Parameter	RAY1216/RTV 300 mg/100 mg BID^*b*^ (*N* = 6)		RAY1216/RTV 500 mg/100 mg BID (*N* = 6)		RAY1216/RTV 700 mg/100 mg BID (*N* = 6)		RAY1216 400 mg TID^*b*^ (*N* = 6)	
	Day 1	Day 5	Day 1	Day 5	Day 1	Day 5	Day 1	Day 5
*t*_1/2_ (h)	3.23 ± 0.286	8.70 ± 1.98	4.48 ± 1.17	9.66 ± 3.26	3.56 ± 0.455	8.31 ± 2.96	2.31 ± 0.465	9.61 ± 2.26
*T*_max_ (h)	1.5 (1.0, 6.0)	2.3 (1.5, 3.0)	1.8 (1.5, 3.0)	2.0 (1.0, 4.0)	2.5 (1.5, 4.0)	1.0 (1.0, 4.0)	1.5 (1.0, 2.0)	1.5 (1.0, 3.0)
*C*_max_ (ng/mL)	4,300 (22.4)	5,940 (26.1)	5,210 (31.8)	8,920 (22.6)	7,390 (27.5)	9,280 (23.2)	3,450 (37.2)	2,700 (32.3)
AUC_0-tau_ (ng·h/mL)	28,300 (30.2)	41,000 (31.8)	36,400 (32)	62,100 (25.6)	48,700 (28)	62,800 (19.1)	10,600 (30.4)	10,000 (36)
AUC_0-t_ (ng·h/mL)	27,300 (29.3)	50,500 (33.1)	36,300 (32)	87,300 (36.3)	48,600 (28.1)	79,900 (20.7)	9,720 (36)	15,400 (32.5)
AUC_0-∞_ (ng·h/mL)	31,900 (32.7)	50,600 (33)	44,600 (26.5)	87,500 (36.3)	55,900 (26)	80,000 (20.6)	13,300 (32)	15,500 (32.2)
*C*_min,ss_ (ng/mL)	–^*c*^	1,180 (47.1)	–	2,520 (47.4)	–	2,150 (36.4)	–	382 (44.9)
V_z_/F (L)	46.8 (25.5)	99.7 (38.8)	79.9 (48.8)	123 (49.2)	69.3 (35.6)	144 (57.2)	110 (39.4)	631 (42.9)
CL/F (L/h)	10.2 (30.4)	8.08 (35.6)	11.9 (27.7)	8.77 (39.5)	13.3 (26.8)	11.5 (19.5)	32.8 (32.3)	44.2 (34.3)
MRT_0-∞_ (h)	6.05 (8.5)	7.60 (14)	7.87 (23)	9.65 (32.2)	6.62 (9.3)	8.09 (10.7)	3.93 (16.5)	6.23 (18.9)
Ra AUC	–	1.46 ± 0.294	–	1.80 ± 0.649	–	1.35 ± 0.356	–	1.04 ± 0.227
Ra *C*_max_	–	1.39 ± 0.241	–	1.91 ± 1.04	–	1.29 ± 0.234	–	0.812 ± 0.220

^*a*^ The mean PK parameters (%CV) are presented except for *t*_1/2_ and Rac (mean and SD) and *T*_max_ (median and range). BID, two times daily; TID, three times daily.

^*b*^ λ_z_ and PK parameters cannot be estimated for the M1007 participants in the RAY1216/RTV 300 mg/100 mg BID group. The corrected determination coefficient *R*_sq, adj,_ M4002 participant in the RAY1216 400 mg TID group was less than 0.8, and the λ_z_ and PK parameters were not included in the statistical analysis.

^*c*^ –, not applicable.

### Excretion and metabolism study

RAY1216 was predominantly found in the plasma, urine, and fecal samples in the form of the parent drug, with relative abundances of 93.38%, 89.61%, and 75.47%, respectively. A total of five metabolites (M7, M15, M17, M18, and M22) were identified. On the basis of the metabolite identification results, we hypothesized that RAY1216 is metabolized primarily *in vivo* through mono-oxidation and hydrogenation pathways, with dehydrogenation serving as a secondary metabolic pathway (Table 5).

**TABLE 5:** Results of metabolites in RAY1216

Metabolite	[M + H]^+^, m/z	Retention time, min	Relative abundance, %								Metabolic pathway
			Plasma				Urine		Fecal		
			0 h–24 h	0.5 h	2 h	8 h	0 h–48 h	0 h–12 h	0 h–96 h	24 h–48 h	
M22	656.3264	34.53	2.38	0.87	2.76	2.92	6.86	6.61	9.21	9.44	Mono-oxidation (P + O)
M7	656.3262	36.50	2.61	1.25	2.75	3.74	2.62	2.95	5.58	5.50	Mono-oxidation (P + O)
M15	642.3473	40.16	0.84	0.25	0.83	1.38	0.42	0.49	8.76	8.71	Hydrogenation (P + 2)
M18	656.3267	41.21	0.68	0.21	0.71	0.98	0.33	0.41	0.33	0.39	Mono-oxidation (P + O)
M17	638.3165	43.93	0.11	0.06	0.14	0.13	0.16	0.13	0.65	0.65	Dehydrogenation (P − 2H)
RAY1216	640.3317	42.67	93.38	97.36	92.81	90.85	89.61	89.40	75.47	75.31	Not applicable

### Tolerability and safety

All AEs were classified as grade 1 or 2 according to the CTCAE 5.0 criteria and were resolved without any treatment measures (see Table 6). There was no significant association between the dose of RAY1216 and the incidence of ADRs; however, the incidence of ADRs was low in the RAY1216 groups, albeit slightly higher than that observed in the placebo group. No grade 3 or above AEs, participant withdrawal due to AEs, or SAEs occurred. There were no significant changes noted in the QTc interphase. These findings indicated a favorable safety and tolerability profile for RAY1216.

**TABLE 6:** ADRs after the administration of RAY1216^*a*^^,^*^b^*

	Single ascending dose and drug-drug interaction						Food effect				Multiple ascending dose				
	RAY1216					Placebo (*N* = 8)	RAY1216 800 mg		RAY1216/RTV 500 mg/100 mg		RAY1216/RTV, BID			RAY1216, TID	Placebo (*N* = 8)
	200 mg (*N* = 6)	200 mg/100 mg RTV (*N* = 6)	400 mg (*N* = 6)	800 mg (*N* = 6)	1,600 mg (*N* = 6)		Fast (*N* = 12)	Fed (*N* = 12)	Fast (*N* = 12)	Fed (*N* = 12)	300 mg/100 mg (*N* = 6)	500 mg/100 mg (*N* = 6)	700 mg/100 mg (*N* = 6)	400 mg (*N* = 6)	
AE^*a*^, *n* (%)	2 (33.3)	2 (33.3)	2 (33.3)	1 (16.7)	2 (33.3)	0	3 (25.0)	5 (41.7)	4 (33.3)	3 (25.0)	3 (50.0)	4 (66.7)	4 (66.7)	2 (33.3)	2 (25.0)
Grade ≥3 AEs	0	0	0	0	0	0	0	0	0	0	0	0	0	0	0
ADR, *n* (%)	2 (33.3)	2 (33.3)	2 (33.3)	0	2 (33.3)	0	2 (16.7)	4 (33.3)	4 (33.3)	3 (25.0)	1 (16.7)	2 (33.3)	3 (50.0)	2 (33.3)	1 (12.5)
Grade 2 ADRs	1 (16.7)	0	0	0	0 (0.0)	0	1 (8.3)	1 (8.3)	1 (8.3)	1 (8.3)	1 (16.7)	0	1 (16.7)	0	0
Hypertriglyceridemia	2 (33.3)	1 (16.7)	0	0	1 (16.7)	0	0	1 (8.3)	3 (25.0)	1 (8.3)	1 (16.7)	1 (16.7)	2 (33.3)	1 (16.7)	0
Hyperuricemia	0	0	0	0	1 (16.7)	0	0	0	1 (8.3)	1 (8.3)	0 (0.0	1 (16.7)	1 (16.7)	0	1 (12.5)
ALT elevated	0	0	0	0	0	0	0	0	0	1 (8.3)	0	1 (16.7)	0	0	0
AST elevated	0	0	0	0	0	0	0	0	1 (8.3)	1 (8.3)	0	1 (16.7)	0	0	0
Bilirubin elevated	0	0	1 (16.7)	0	1 (16.7)	0	0	0	0	0	0	0	0	0	0
Serum creatinine elevated	0	0	1 (16.7)	0	0	0	0	2 (16.7)	1 (8.3)	1 (8.3)	0	0	1 (16.7)	0	0
Neutrophil decreased	0	0	0	0	0	0	1 (8.3)	0	0	0	0	0	0	0	0
Leukopenia	0	0	0	0	0	0	1 (8.3)	0	0	0	0	0	0	0	0
Fatigue	0	0	0	0	0	0	0	0	0	0	0	1 (16.7)	0	0	0
Nausea	0	0	0	0	0	0	0	0	0	0	0	1 (16.7)	0	0	0
Diarrhea	0	0	0	0	0	0	0	0	0	0	0	1 (16.7)	0	0	0
Palpitations	0	1 (16.7)	0	0	0	0	0	0	0	0	0	1 (16.7)	0	0	0
Anemia	0	0	0	0	0	0	0	0	0	0	0	1 (16.7)	0	0	0

^*a*^ All AEs were classified by system organ category, and the terms of the newest version of MedDRA were used. The CTCAE 5.0 was used to grade the AEs (grades 1–5). No grade ≥3 AEs were observed.

^*b*^ ADR, adverse drug reaction; *N*, number of participants analyzed; *n*, number of participants who experienced at least one AE; BID, twice daily; TID, three times daily; RTV, ritonavir.

In the SAD of RAY1216, 6 out of 32 participants (18.8%) reported at least one ADR, with these participants receiving varying doses of RAY1216: two participants (33.3%) in each of the 200 mg, 400 mg, and 1,600 mg cohorts. In the DDI study of RAY1216 in combination with ritonavir, two participants (25%) experienced at least one ADR.

In the food-effect study of RAY1216, 5 out of 12 participants (41.7%) experienced at least one ADR. Among these, 2 out of 12 participants (16.7%) experienced reactions while fasting, whereas 4 out of 12 participants (33.3%) experienced reactions after meals. Furthermore, in the food-effect study of RAY1216 in combination with ritonavir, 4 out of 12 participants (33.3%) reported ADRs, with 4 out of 12 participants (33.3%) experiencing ADRs while fasting and 3 out of 12 participants (25.0%) experiencing ADRs after meals.

In the MAD study investigating RAY1216 with or without ritonavir, 9 out of 32 participants (28.1%) reported at least one ADR. Specifically, one participant (12.5%) in the placebo group, one participant (16.7%) in the RAY1216/RTV 300 mg/100 mg group, two participants (33.3%) in the RAY1216/RTV 500 mg/100 mg group, three participants (50.0%) in the RAY1216/RTV 700 mg/100 mg group, and two participants (33.3%) in the RAY1216 400 mg TID group experienced ADRs.

The most frequently reported ADRs included hypertriglyceridemia, anemia, hyperuricemia, and transient elevation of serum creatinine levels.

**Fig 3:**
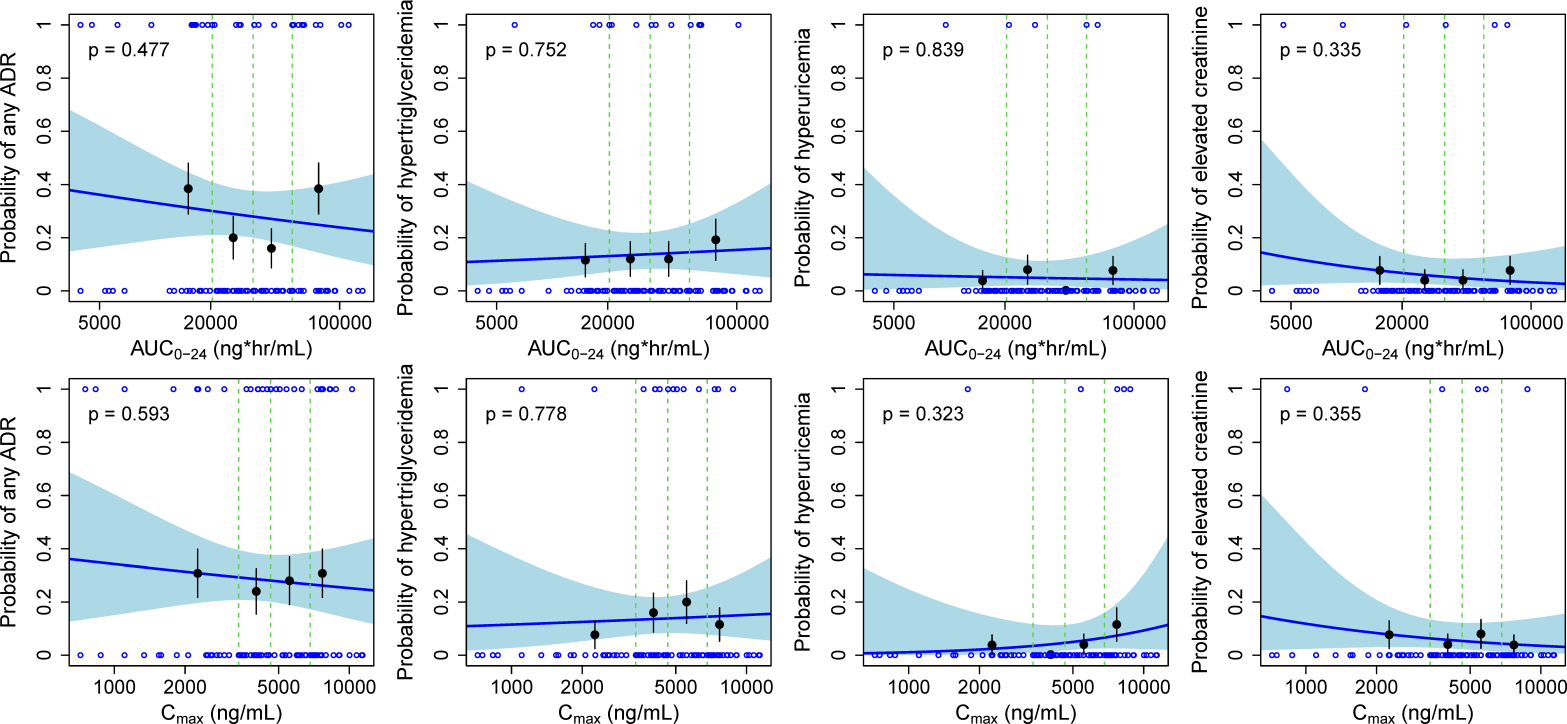
The open blue circles reflect the observed adverse events. The filled black symbols are the observed probability of events, and the error bars are SEs [sqrt (P * (1 P)/4)] for quantiles (at 100×(1/4)th percentiles) of exposures (plotted at the median value within each quantile). The blue line and the light blue shaded area represent the average model-predicted probability and the 95% prediction interval, respectively.

The results of the exposure-response (E-R) analysis indicated that there was no significant dose-response relationship between ADRs, hypertriglyceridemia, hyperuricemia, or elevated creatinine levels and exposure. The results of the experiment are shown in Fig. 3.

No participant exhibited significant changes in the evaluation indices of the QTc interval. One participant in the placebo group and one in the RAY1216 700 mg combined with ritonavir BID group each displayed an absolute value of QTcF exceeding 450 ms. However, no participant had an absolute QTcF greater than 470 ms in men or greater than 480 ms in women. Ten participants had a ΔQTcF greater than 30 ms, with no participants exceeding a ΔQTcF of 60 ms. C-QT model analysis indicated no significant correlation between drug concentration and the QT interval. The model predicted that the upper limit of the 90% CI for the average ΔΔQTcF at the average *C*_max_ concentration in each cohort was less than 10 ms. In the group receiving the highest dose (RAY1216 700 mg combined with ritonavir BID), the mean ΔΔQTcF at an average *C*_max_ concentration of 9280 ng/mL was 2.6819 ms (90% CI: −1.6526–7.0164), suggesting that RAY1216 had no clinical effect on the QTcF interval.

## DISCUSSION

To date, multiple antiviral drugs have been approved to address the ongoing threat posed by COVID-19 (24). The experimental drug examined in this study, i.e., RAY1216, is one of the approved antiviral agents. RAY1216, named “leritrelvir,” has been approved as a single-component drug for treating COVID-19 in China.

In the present study, we first reported the PK characteristics, safety, and tolerability of the SARS-CoV-2 3CL protease inhibitor RAY1216 in healthy Chinese adult participants. The safety results showed that a single ascending dose (from 200 to 1,600 mg) of RAY1216 alone or combined with 100 mg RTV and a multiple ascending dose (from 300 to 700 mg) of RAY1216 alone or combined with 100 mg RTV were safe and well tolerated among healthy participants. The most commonly reported ADRs were hypertriglyceridemia, hyperuricemia, and elevated levels of serum creatinine. There was no significant correlation between these ADRs and drug exposure according to the results of E-R analysis. RAY1216 was well-absorbed after administration with exposure increasing in a dose-dependent manner. Food appeared to increase exposure to and delay the absorption of RAY1216. Ritonavir significantly inhibits drug metabolism and increases drug exposure. Preclinical pharmacodynamic evaluations have shown that the anti-COVID-19 activity of RAY1216 is comparable to that of nirmatrelvir (18). These results support the need for phase II/III studies in participants with mild-to-moderate COVID-19.

In the SAD study, the dose-dependent increase in CL/F can be attributed to differences in bioavailability between individuals, as the volume of distribution also increased. In addition, the MAD study revealed a dose-dependent increase in exposure on day 1 but a lower dose-dependent increase in exposure on day 5. Given the small sample size and considerable interindividual variability, this change did not reach statistical significance. There was a slight accumulation of RAY1216 when it was administered in combination with ritonavir; however, no accumulation was observed after multiple administrations of 400 mg of RAY1216 TID. The duration of anti-COVID-19 treatment was short (5 days), and the accumulation of the drug following continuous administration was not clinically significant (25–27).

The DDI study showed that ritonavir can significantly increase the plasma exposure of RAY1216 without increasing the associated safety risks. Hence, ritonavir can be a PK enhancer and can be co-administered with RAY1216 consistent with nirmatrelvir (13).

Following consecutive treatment with RAY1216/ritonavir (300 mg/100 mg BID) and RAY1216 400 mg TID at steady state, the mean trough concentrations (*C*_min,ss_) were observed to be 1,180 ng/mL and 382 ng/mL, respectively. These concentrations are sufficient to surpass the antiviral 90% effective concentration (EC_90_) value (wild-type strain EC_90_, 165 ng/mL; Omicron EC_90_, 209.7 ng/mL, corrected by the plasma protein binding rate) *in vitro*. It is challenging to achieve the EC_90_ with nirmatrelvir alone. These findings suggest that the PK characteristics of RAY1216 alone are superior to those of nirmatrelvir (17, 25). Preclinical studies have indicated that single mutants can reduce RAY1216 and nirmatrelvir inhibition to similar degrees. However, compared with nirmatrelvir, two mutants (E166A/L167F and L50F/E166V) had a weaker effect on RAY1216 inhibition, and RAY1216 had a longer drug-target residence time and dissociated from Mpro at a slower rate (18). Although the steady-state trough concentration at the clinical dose of nirmatrelvir/ritonavir was above its EC_90_ value, the use of RAY1216 alone rather than in combination with ritonavir offers greater convenience and minimizes the risks associated with multiple drug interactions (24, 25). Consequently, the use of RAY1216 alone may also be regarded as a viable dosing regimen.

The absorption of RAY1216 was delayed and slightly increased after a high-fat meal, a phenomenon also observed with similar target drugs (17, 28). Importantly, this did not impact the safety profile of the drug, allowing for RAY1216 to be administered without meal restrictions.

In this study, RAY1216 was well tolerated and demonstrated safety in healthy participants. Oral doses of RAY1216 ranging from 200 to 1,600 mg were found to be safe and well tolerated by healthy volunteers. All the reported AEs were classified as mild or moderate. The AEs observed in our study align with those reported in previous studies of similar drugs, supporting findings from analogous Japanese research that evaluated the safety and PK of ensitrelvir in healthy participants (28).

While our current trial has enhanced the understanding of the PK, safety, and mode of action of RAY1216, it is important to acknowledge certain limitations. For this trial, we exclusively enrolled healthy participants, and the sample size was relatively small. Consequently, it will be crucial to include a larger population in future phase 2 and 3 clinical trials.

During the trial, the analysis was conducted in a progressive manner. On the basis of preclinical results and the evaluation of the PK and safety profiles of RAY1216, the combination of 200 mg or 300 mg RAY1216 with a 100 mg ritonavir BID regimen would be adequate. However, for RAY1216 monotherapy, a minimum dosage of 400 mg TID was necessary to achieve the desired target concentrations. Ultimately, on the basis of population PK analysis and simulation, a dosing regimen of 400 mg TID was selected (23). These PK and safety data offer a rapid and robust foundation for subsequent dose selection. Furthermore, the findings of this study are highly important for future research and clinical applications of RAY1216, particularly in the exploration of drug interactions.

## Data Availability

The data that support the findings of this study are available from the corresponding author upon reasonable request. All the data generated or analyzed during this study are included in this published article. However, individual participant-level raw data containing identifiable subject information cannot be shared.
